# A New Caustic for Toothache

**Published:** 1862-01

**Authors:** 


A New Caustic for Toothache.—The following treatment is recommended by Dr. Calvy, of Toulon, for the neuralgia proceeding from carious teeth. The carious cavity is first to be cleaned out, and then a piece of cotton, steeped in a solution of six grains of acetate morphia in an ounce of nitric acid, is to be applied to its interior. As soon as the caustic penetrates into the carious tooth, the pain ceases, and the patient is cured. After the application of the caustic, the cavity should be filled with cotton steeped in the sedative solution of opium, and afterwards permanently plugged.—*Br. Med. Jour.* from *Gaz. des Hospitaux.*

This is rather heroic treatment, and we think would make quite a stir if applied as directed.

COLUMBUS, OHIO, Dec. 26th, 1861.

POSTPONEMENT.

AFTER consultation with most of the members of the Central Ohio Dental Association, it is thought best to postpone the next meeting of this Society until the national troubles shall have subsided, or until called together by the President.

J. G. HAMILL, President.

THOS. M'CUNE, Corresponding Secretary.

